# New Books

**Published:** 2006-06

Academic Scientists at Work, 2nd ed.

Jeremy Boss, Susan Eckert

New York:Springer, 2006. 296 pp. ISBN: 0-387-32176-4, $29.95

Advances in Knowledge Discovery and Data Mining

Wee Keong Ng, Masaru Kitsuregawa, Jianzhong Li, eds.

Berlin:Springer-Verlag, 2006. 879 pp. ISBN: 3-540-33206-5, $119

Amazing Numbers in Biology

Rainer Flindt

Berlin:Springer-Verlag, 2006. 296 pp. ISBN: 3-540-30146-1, $29

Antifouling Compounds

Nobuhiro Fusetani, Anthony S. Clare, Werner E.G. Müller, eds.

Berlin:Springer-Verlag, 2006. 225 pp. ISBN: 3-540-30014-7, $159

Bird Flu: A Rising Pandemic in Asia and Beyond?

Paul A Tambyah, Ping-Chung Leung

Hackensack, NJ:World Scientific Publishing Co., 2006. 268 pp. ISBN 981-256-815-8, $48

Fundamental Toxicology

J. H. Duffus, H. G. J. Worth, Eds.

New York:Springer, 2006. 462 pp. ISBN: 0-85404-614-3, $89.95

Gene Mapping, Discovery, and Expression: Methods and Protocols

Minou Bina, ed.

Totowa, NJ:Humana Press, 2006. 352 pp. ISBN: 1-58829-575-3, $99.50

Genomic Disorders: The Genomic Basis of Disease

James R. Lupski, Pawel T. Stankiewicz

Totowa, NJ:Humana Press, 2006. 550 pp. ISBN: 1-58829-559-1, $130.50

Global Coastal Change

Ivan Valiela

Williston, VT:Blackwell Publishing, 2006. 376 pp. ISBN: 1-405-13685-5, $89.95

Global Warming: Understanding the Forecast

David Archer

Williston, VT:Blackwell Publishing, 2006. 256 pp. ISBN: 1-405-14039-9, $49.95

Globalization and Health: Challenges for Health Law and Bioethics

Belinda Bennett, George F. Tomossy

New York:Springer, 2006. 218 pp. ISBN: 1-4020-4195-0, $159

Globalization and Its Enemies

Daniel Cohen

Cambridge, MA:MIT Press, 2006. 256 pp. ISBN: 0-262-03350-X, $27.95

Groundwater in the Environment

Paul Younger

Williston, VT:Blackwell Publishing, 2006. 224 pp. ISBN: 1-405-12143-2, $45

Information Visualization: Beyond the Horizon

Chaomei Chen

New York:Springer, 2006. 320 pp. ISBN: 1-84628-340-X, $49.95

Managing Nano-Bio-Info-Cogno Innovations: Converging Technologies in Society

William Sims Bainbridge, Mihail C. Roco, eds.

New York:Springer, 2006. 390 pp. ISBN: 1-4020-4106-3, $129

Math Refresher for Scientists and Engineers, 3rd ed.

John R. Fanchi

Indianapolis, IN;Wiley-IEEE Press, 2006. 364 pp. ISBN: 0-471-75715-2, $59.95

Organic Contaminants in Riverine and Groundwater Systems

Jan Schwarzbauer

Berlin:Springer-Verlag, 2006. 464 pp. ISBN: 3-540-31169-6, $179

Sustainable Metals Management

Arnim von Gleich, Robert U. Ayres, Stefan Gößling-Reisemann, eds.

New York:Springer, 2006. 607 pp. ISBN: 1-4020-4007-5, $209

Transactions on Computational Systems Biology IV

Corrado Priami, Luca Cardelli, Stephen Emmott, eds.

Berlin:Springer-Verlag, 2006. 141 pp. ISBN: 3-540-33245-6, $64.95

